# Methylation Dynamics in *Helicobacter pylori*: Exploring Acidic Stress Effects on Epigenetic Acclimation

**DOI:** 10.3390/microorganisms14071501

**Published:** 2026-07-09

**Authors:** Sarah K. Patterson, Joanna Y. He, Yixin Xu, Ella M. Greene, Yaroslav Poznyak, Mary Virginia Nye, Mark H. Forsyth

**Affiliations:** Department of Biology, William & Mary, 540 Landrum Drive, Williamsburg, VA 23187, USA; skpatterson@wm.edu (S.K.P.); jyhe@wm.edu (J.Y.H.); monicaxu2027@u.northwestern.edu (Y.X.); egreene@wm.edu (E.M.G.); ypoznyak@wm.edu (Y.P.); mn3297@columbia.edu (M.V.N.)

**Keywords:** *Helicobacter pylori*, DNA methylation, two-component systems, acid acclimation

## Abstract

*Helicobacter pylori* possesses an unusually high number of restriction–modification (R-M) systems relative to its small genome, contributing to a methylome increasingly implicated in bacterial gene regulation. In this study, we analyzed the methylomes of two mutant strains of *H. pylori* 26695: ∆*rdxA* (control) and ∆*rdxA*/∆*arsS*. Each mutant was cultivated under neutral (pH 7) and acidic (pH 5) growth conditions. We identified one conspicuous hypomethylated region of 21 kBp possessing 21 annotated genes across each methylome. Notably, over 600 protein coding regions and 10 different promoters displayed differential methylation between pH conditions, including several virulence factors. The *vacA* gene, encoding the Vacuolating Cytotoxin A, exhibited eight differentially methylated positions between pH 7 and pH 5 within the *H. pylori* 26695 control mutant methylome, potentially contributing to its previously documented 32-fold down regulation of mRNA in acidic environments. pH-dependent methylation changes were widespread within the *cag* pathogenicity island, genes encoding cell envelope proteins including adhesin-encoding *sabA, babA*, and *hopQ,* and numerous flagellar-associated genes. These results reveal the plasticity of the *H. pylori* methylome and suggest that DNA methylation is responsive to environmental pH in both ArsRS-dependent and independent manners. Methylome dynamics may serve as an important layer of gene regulation in acclimation to hostile gastric environments and promote persistent infection.

## 1. Introduction

*Helicobacter pylori* is a Gram-negative, spiral-shaped bacterium that colonizes the gastric mucosa of over half of the global population [1,2,3] and is a well-established risk factor for atrophic gastritis, gastric cancer, gastric lymphoma and both gastric and duodenal ulcers [4,5,6,7,8]. Despite the highly acidic and fluctuating environment of the stomach, as well as robust host inflammatory and immune responses, *H. pylori* is able to persistently infect due to effective acclimation and adaptation to the gastric environment [9,10,11].

Studies from our lab and others have shown DNA methylation plays a crucial role in regulating *H. pylori* gene expression by adding a methyl group to specific cytosine or adenine nucleotide bases [12,13,14,15,16]. DNA methylation can modulate gene expression by altering DNA interaction with RNA polymerase, and/or regulatory proteins, control DNA replication by impacting the initiation, termination, and fidelity of the process, and shape cellular phenotypes by driving phenotypic diversity [17,18,19]. Bacterial DNA methylation has historically been studied in the context of restriction–modification (RM) systems, where its primary role is to protect the cell from invading DNA by differentiating between the host’s own methylated DNA and foreign, non-methylated DNA [20]. RM systems may also play a role in regulating gene expression [15,21,22,23]. RM systems typically comprise two components: a methyltransferase (MTase) that adds a methyl group to a specific DNA sequence motif and a restriction endonuclease (REase) that cleaves double-stranded DNA at the same motif if it lacks the protective methylation [20,23]. A number of genomes are also known to encode MTases that are not associated with an REase and are termed “orphan/solitary MTases” [23]. RM systems in *H. pylori* are of particular interest due to the relatively small size of the bacterial genome (~1.6 million base pairs) but relatively high abundance of RM systems (20–30) [15,24]. Despite this high abundance, many RM genes are inactive, and strain-specific RM genes account for 15–20% of genetic differences between closely related *H. pylori* strains such as 26695 and J99 [25]. Notably, while 90% of RM genes are shared between these strains, the active RM genes tend to be strain-specific rather than conserved [25].

Due to the importance of managing the acidic environment of the host stomach to *H. pylori*, we sought, for the first time, to elucidate a relationship between DNA methylation and conditions of extracellular acidity. We hypothesized that dynamic epigenetic changes may occur as a result of environmental changes, as another potential layer of regulation of gene expression in *H. pylori* in its acclimation to acidity changes. Additionally, we sought to determine whether any acid-induced changes in the methylome were dependent upon a major acid-sensing two-component system (TCS) in *H. pylori*—the acid-responsive signaling sensor (ArsS) and response regulator (ArsR) [26,27,28,29,30]. Previously, we demonstrated that the Type I methyltransferase HsdM1 (HP0463) is transcriptionally regulated by the ArsRS TCS and conditions of acidity [12]. In the course of that study, we determined the methylomes of *H. pylori* 26695 grown at either pH 7 or pH 5, with and without a functional ArsRS TCS (Table 1). In the current study, we extensively analyze the patterns and differences observed across each of the four methylomes determined in our previous study. We characterize methylome changes in vitro in *H. pylori* strain 26695 using SMRT DNA sequencing of *H. pylori* genomes harvested from cells cultured under four experimental conditions listed in Table 1: cultivation at pH 7, at pH 5, and at each of these pH values in the absence of a functional ArsRS TCS [12]. Notably, SMRT sequencing can detect a variety of DNA modifications. While our data called for m6A, m4C, m5C, and modified bases, the final analyses focused exclusively on m6A and m4C methylations due to their higher reliability. The infrequent SMRT sequencing calls of m5C methyl marks are known to be unreliable with this technique. Additionally, the modifications of the modified bases called may include methylation, formylation, carboxylation, and other types of modifications, but these were excluded for the purpose of this study [31,32].

Some methylation patterns observed in *H. pylori* 26695 suggest conserved methylome organization regardless of culture pH or a functional ArsRS TCS. This is in contrast to numerous genes that exhibit differential methylation within promoters or coding regions depending on the culture pH or the presence/absence of a functional ArsRS TCS, indicating regulatory plasticity in response to external stimuli. The influence of both acidity and the ArsRS TCS on these methylation changes underscores a dynamic role for restriction–modification systems beyond their traditional function, potentially contributing to environmental acclimation in *H. pylori*.

## 2. Materials and Methods

### 2.1. Helicobacter pylori Strains and Culture Conditions

The culture of *H. pylori* strains and the construction of the *arsS* null mutant were described in our earlier studies [12,30]. Briefly, *H. pylori* strains were cultured on Trypticase Soy Agar II plates (BD BBL, Franklin Lakes, NJ, USA) with 5% sheep blood at 37 °C in 5% CO_2_/95% ambient air or in Sulfite-free Brucella broth supplemented with a Cholesterol-Lipid supplement (Gibco, Thermo Fisher Scientific, Waltham, MA, USA). The *H. pylori* 26695 Δ*rdxA* strain, a metronidazole-resistant mutant, was used as the control strain. The Δ*rdxA* mutant served as the basis for constructing the Δ*arsS* strain using a counter-selection procedure described in Loh et al. [33]. This controlled for any potential effects of the Δ*rdxA* locus, which encodes an oxygen-insensitive NADPH nitroreductase unrelated to acid response.

### 2.2. Methylome Experiment

To investigate the effects of acid exposure on the ArsRS system and the *H. pylori* methylome, long-term acid exposure experiments were conducted on the control mutant and Δ*arsS*. Both control and isogenic *arsS* deletion mutants of *H. pylori* 26695 were initially grown overnight in Sulfite-free Brucella broth pH 7 that was supplemented with a Cholesterol-Lipid supplement (Gibco) from 24 to 36 h 5% sheep blood agar plates. Each mutant was subcultured in the same medium to an OD600 of 0.2. Once the cell reached mid-logarithmic growth (OD_600_~0.8–1.0), equal aliquots were harvested by centrifugation, the supernatant was discarded and cells were resuspended in the same volume of Sulfite-free Brucella broth at either pH 7 or pH 5 and grown overnight. Cells were passed to fresh medium at either pH 7 or pH 5 to an initial OD_600_ of 0.2 and grown overnight. This was repeated until cells had grown for 72 h under either pH condition before harvesting for SMRT sequencing. Bacterial cells were collected via centrifugation and stored at −20 °C before DNA extraction. A detailed description of the culture conditions and experimental procedures can be found in our previous study [12].

### 2.3. Methylome Sequencing

Genomic DNA was extracted using the Bio-Rad genomic DNA extraction kit (Hercules, CA, USA), following the manufacturer’s protocol with RNA elimination. DNA quality and concentration were assessed using a Nanodrop (Thermo Fisher, Waltham, MA, USA), and integrity was confirmed via agarose gel electrophoresis. Whole-genome methylome sequencing was performed by Azenta Life Sciences (Burlington, MA, USA) using the PacBio Sequel platform (Pacific Biosciences, Menlo Park, CA, USA), which detects N6-methyladenosine, N4-methylcytosine, and N5-methylcytosine modifications via Single Molecule, Real-Time (SMRT) sequencing. A detailed description of the sequencing can be found in our previous publication [12].

### 2.4. Bioinformatic Analysis of Methylation Data

PacBio methylome sequencing generated GFF files containing key information for each methylation site, including the sample ID, base-pair position, strand orientation (+/−), local sequence context (20 base pairs upstream and downstream, totaling 41 base pairs), motif, IPD ratio, and coverage. The score represents confidence in methylation detection, while coverage refers to the number of sequencing reads supporting the methylation call.

Data processing and analysis were conducted using Python (version 3.12) with the following libraries: Pandas (2.2.3), GffPandas (1.1.2), and NumPy (2.2.4). The GFF files were loaded into Python as Pandas dataframes, allowing for efficient data organization and filtering. To ensure high-confidence methylation calls, only methylation sites with an IPD ratio ≥ 2 were retained.

### 2.5. Mapping Methylation Sites to Genomic Features

To quantify methylation within coding regions, we used the annotated genome from Tomb et al., 1997 defining coding regions as methylation sites occurring between the translational start and end positions of each gene [24]. Each methylation site was assigned to its corresponding coding region ID within the dataframe.

For promoter region methylation, transcriptional start sites (TSSs) were obtained from Sharma et al. (2010) using exclusively primary promoters [34]. Promoter regions were defined as the 50 base pairs upstream of the transcription start site (TSS − 50) for positive-strand genes, and the 50 base pairs downstream of the TSS (TSS + 50) for negative-strand genes. A separate data frame containing TSS positions and strand orientation was used to count the number of methylation events occurring within these defined promoter regions.

### 2.6. Defining Hypermethylation and Data Visualization

**Figure 1:**
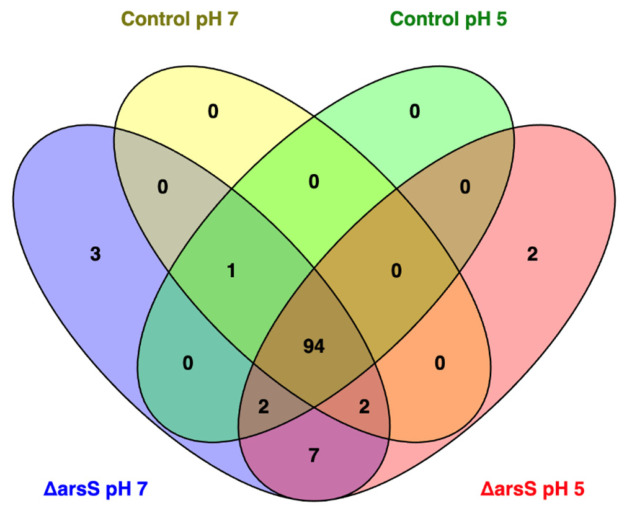
**The relationship between hypomethylation, culture pH, and the ArsRS two-component system.** A Venn diagram represents the genes identified as hypomethylated for each methylome. Hypomethylated genes consistent between methylomes are displayed as overlapping ellipses.

To identify hypermethylated promoters and coding regions, we used a method established by Mou et al. [35], classifying regions with methylation frequencies ≥ 3 standard deviations above the mean as hypermethylated. The histograms in Figure 1 were generated using custom R code (version 2024.02.2+764) provided by Mou’s lab and can be located within the Forsyth GitHub (version 3.21.2).

### 2.7. Genome-Wide Classification of Methylation Events

To categorize each methylation site as occurring in a coding region, promoter, both, or intergenic region, we created a genomic annotation array for the length of *H. pylori*’s genome (1,667,867 base pairs). Promoter regions were indexed first, with 1 assigned to promoter positions. Coding regions were indexed next, with 2 assigned to gene positions—ensuring that if a promoter site “1” overlapped a coding region, it was instead marked as “3” to denote an overlapping promoter and coding region. All remaining positions were designated as “0” (intergenic regions).

Each methylation site from the PacBio GFF files were then mapped to this array, categorizing it as promoter, “1,” coding region, “2”, both, “3”, or intergenic, “0”. The corresponding *H. pylori* 26695 gene locus tag was appended to each methylation site, facilitating functional analysis.

## 3. Results

### 3.1. Study Design

Our control mutant, *H. pylori* 26695 Δ*rdxA*, possesses a wild-type *arsRS* locus. The deletion of the nitroreductase gene *rdxA* allows for the generation of markerless mutations elsewhere in the genome using a counter-selection method [33]. We also used an isogenic *arsS* deletion mutant, allowing for the investigation of the role of this acid-sensing histidine kinase in DNA methylation as a response to acidic environments [30]. To investigate the impact of pH on *H. pylori* DNA methylation, we cultured the strains under two different pH conditions: pH 7 (neutral) and pH 5 (acidic), resulting in four experimental groups (Table 1).

### 3.2. The H. pylori 26695 Genome Possesses an Undermethylated Region Containing Genes Associated with Mobile DNA

The role of DNA hypomethylation in bacterial gene expression is not well-understood. Some hypothesize that the presence of a methyl group on adenine or cytosine affects the interaction of RNA polymerase with promoters, thereby altering transcription [36]. To investigate hypomethylation patterns, we tested a hypothesis that variation in methylated DNA due to pH was dependent upon ArsRS, a well-documented control factor in acid acclimation.

**Figure 2:**
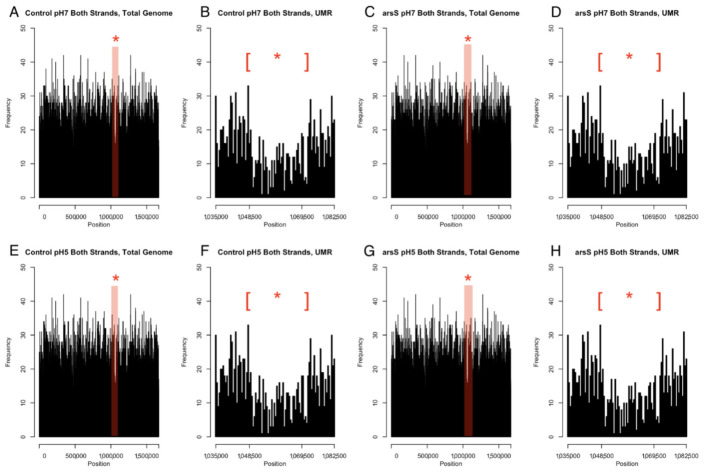
**Genome-wide methylation analysis identifies an undermethylated region (UMR) in the *H. pylori* 26695 genome.** Histogram plots for the methylation counts of each sample are depicted. The frequency of methylation was summed for 500 base-pair bins to determine the relative distribution of methylation across the genome of *H. pylori* 26695, in both a control (**A**,**B**,**E**,**F**) and an *arsS* null mutant (**C**,**D**,**G**,**H**) cultured at pH 7 (**A**–**D**) or pH 5 (**E**–**H**). * Undermethylated region (UMR) relative to the rest of the methylome.

We analyzed methylation patterns across the *H. pylori* 26695 methylomes determined in our 2024 study [12] (Table 1) using 500 base-pair bins. On average, 3.56% of all nucleotides in the control methylomes were methylated, and only slightly less at 3.52% were methylated in the Δ*arsS* samples. These percentages were determined using the total number of methyl marks in the *H. pylori* 26695 control methylome (59,428 methylations at pH 7 and 59,416 at pH 5) and in the *arsS* deletion mutant methylome (58,674 methylations at pH 7 and 58,641 at pH 5) as percentages of the total number of base pairs in the genome, 1,667,867 bp. This analysis revealed a consistently undermethylated region (UMR) of greater than 21 kB approximately between base pairs 1,048,000 and 1,069,000. This UMR was characterized by fewer than 10 methylations per 500 base-pair bins, corresponding to a methylation frequency of 2% or less. The UMR was similarly undermethylated across all four experimental conditions (control and Δ*arsS* mutants cultivated at pH 7 and pH 5; Figure 2).

The UMR of *H. pylori* strain 26695 possesses 21 annotated genes between HP0985 and HP1006, encoding 15 hypothetical proteins, as well as IS605 transposase A & B, integrase/recombinase (*xerD*), PARA protein, and a predicted conjugal transfer protein (*traG*). The presence of genes predicted to be involved in mobile DNA elements in the UMR may suggest that horizontal gene transfer may be affected by methylation status. DNA methylation varies only slightly within this UMR as a function of pH or ArsRS TCS status. Comparing the *H. pylori* 26695 control methylomes grown at pH 7 and pH 5, the methyl counts within the UMR were 450 and 451, respectively. Analysis of this same region within the Δ*arsS* mutants cultured at pH 7 and pH 5 revealed methylation counts of 442 and 443, respectively.

### 3.3. Cag Pathogenicity Island Genes cag2, cag12, and cag15 Are Hypomethylated

Hypomethylated genes are of interest, as the lack of methyl groups may alter gene expression. Using < 2% as a threshold to define hypomethylation, we extended our analysis to identify individual hypomethylated genes throughout the genome. Notably, not all identified hypomethylated genes were confined to this 1,048,000–1,069,000 bp UMR illustrated in Figure 2. While this undermethylated region was shared across methylomes regardless of the pH of the culture or the functional status of the ArsRS TCS, variations in hypomethylation at the individual gene level elsewhere in the genome were influenced by pH conditions and the presence or absence of a functional ArsRS system. This gene-to-gene variation in methylation based on pH or ArsRS status throughout the genome suggests that epigenetic factors may dynamically shape *H. pylori* gene expression as a means of acclimation to changes in the environment.

A total of 94 genes, including many of the 21 genes of the UMR, were hypomethylated across all four studied methylomes (Figure 1). Hypomethylated genes outside the UMR included HP0091, encoding a type II restriction enzyme (*hsdR*), HP0481, encoding an adenine-specific DNA methyltransferase, and HP0676, encoding methylated-DNA–protein-cysteine methyltransferase. Each of these DNA restriction–modification system genes were consistently hypomethylated in each of the methylomes determined in our studies. Virulence-associated genes, such as HP0315 (*vapD*) and HP0459, a *virB*4 homolog, were hypomethylated across all methylomes regardless of pH or ArsRS status, as was HP0613, encoding an ABC transporter ATP-binding protein. Additionally, hypomethylation of the ulcer-associated gene encoding the restriction endonuclease IceA (HP1209) and *cag*PAI components, HP0521 (*cag2*), HP0533 (*cag12*) and HP0536 (*cag15*), suggest possible involvement of hypomethylated DNA in the proper expression of these virulence factors. The shared nature of hypomethylation among these genes may represent conserved mechanisms important for *H. pylori*’s survival and virulence regardless of pH conditions. The remaining hypomethylated genes outside the UMR encode hypothetical proteins.

Our analysis also identified distinct patterns of gene hypomethylation across experimental conditions. In the Δ*arsS* mutant cultivated at pH 7, three genes showed unique hypomethylation; HP0627 and HP1518, encoding proteins of unknown function, and superoxide dismutase (*sodB*-HP0329). At pH 5, the Δ*arsS* mutant exhibited unique hypomethylation of the hypothetical protein encoding gene HP0482 and glucose-6-phosphate isomerase (pgi) (HP1166). The *H. pylori* 26695 Δ*arsS* mutant possessed a slightly higher total number of hypomethylated genes, 109 at pH 7 and 107 at pH 5, relative to the 97 hypomethylated genes within the isogenic control methylomes discussed above. This is an indication that environmental pH may affect epigenetic changes in the *H. pylori* 26695 genome that are independent from the ArsRS TCS.

Six genes showed consistent hypomethylation in the Δ*arsS* null mutants regardless of pH; HP0053, HP0078, HP0346, HP0996, the multidrug resistance protein SpaB encoding gene (HP0600), and HP1249. In the *H. pylori* 26695 control methylomes, each of these genes possessed increased levels of methylation that was independent of the pH at which they were grown. This pattern suggests direct ArsRS-dependent regulation of the methylation of these genes independent of environmental pH.

There were no genes that were hypomethylated only within the *H. pylori* 26695 control methylome compared to the Δ*arsS* pH 7 methylome. HP0423 and HP1085, encoding hypothetical proteins, were hypomethylated in the control pH 7 methylome, and both Δ*arsS* methylomes, but not within that of the control pH 5 methylome. Two hypothetical protein encoding genes, HP0174 and HP1590, were hypomethylated in three conditions: *arsS* null mutant at both pH 7 and pH 5, and the control strain at pH 5, suggesting these genes may be particularly sensitive to environmental pH in an ArsRS-independent manner.

### 3.4. Greater than 50% of H. pylori 26695 Promoters Are Unmethylated

Across the four *H. pylori* methylomes analyzed in our studies, a large number of genes and operons exhibit an absence of methylation in their annotated promoter regions. Of 816 primary promoters of *H. pylori* strain 26695 annotated by Sharma and colleagues in their 2010 study [34] (defined as the transcriptional start site plus 50 bp upstream), we determined that nearly 54% (441) were consistently unmethylated across all four *H. pylori* 26695 methylomes characterized in this study. In the control *H. pylori* 26695 mutant, 443 primary promoters were unmethylated at pH 7 and 445 at pH 5. Similarly, in the Δ*arsS* mutant, 446 and 447 gene promoters were unmethylated at pH 7 and pH 5, respectively. Although most of these promoters are associated with genes that encode hypothetical proteins, the second most prevalent classification for the genes driven by these unmethylated promoters encode products involved with the cell envelope. Our analysis also found that the majority of the unmethylated promoters are unaffected by pH or the functional status of ArsRS TCS (Supplemental Table S1).

The annotated promoter region of HP0543 (*cag22*) exhibited a distinctive methylation pattern dependent upon both culture pH and the ArsRS TCS. In the *H. pylori* 26695 control strain possessing an intact ArsRS, this promoter was methylated when cells were cultured at pH 7 but unmethylated when grown under acidic conditions (pH 5). In contrast, in the *arsS* null mutant, this promoter remained methylated regardless of environmental pH. This methylation pattern suggests that the ArsRS system actively regulates methylation of this *cag*PAI promoter in response to pH changes, potentially as part of the *H. pylori* acid acclimation mechanism. There is no direct evidence that *cag22* is involved in virulence, as it was shown to be dispensable for CagA translocation and IL-8 induction [37]. However, since its role in the *cag*PAI T4SS is as yet unknown, this pH-dependent, ArsRS-mediated regulation of promoter methylation potentially suggests that it plays a role in *H. pylori*’s response to the acidic gastric environment and modulates its expression accordingly.

Our analysis identified promoters uniquely methylated under different conditions, potentially shedding light on the environmental acclimation of the *H. pylori* epigenome and the impact of the ArsRS regulatory system. When cultivated at pH 7, but not when cultivated at pH 5, the *H. pylori* 26695 control mutant possessed unique methylation marks within promoter regions for four genes: HP0222 (*copG*), HP0777 (*pryH*), HP1399 (*rocF*), and HP0408 (hypothetical). RocF is an arginase crucial for acid protection, and *pyrH* encodes UMP kinase that is required for nucleotide synthesis. Under acidic (pH 5), but not neutral, growth conditions, the control mutant possesses additional methylations in only the promoter of HP0408 (hypothetical).

We also identified three promoters methylated in the control mutant and unmethylated in the *arsS* deletion mutant at both pH conditions: HP0163 (δ-aminolevulinic acid dehydratase), HP1066 (hypothetical protein), and the regulatory RNA *HPt19*. Thus, methylation of these promoters appears to be ArsRS-dependent, but independent of pH. Our analysis found no promoters uniquely methylated within the Δ*arsS* mutant at pH 5. The methylation of these three gene promoters exclusively in the *H. pylori* 26695 control mutant suggests that the ArsRS system is essential for maintaining methylation at these regulatory regions.

### 3.5. Hypermethylated Promoters Are Unaffected by pH or ArsRS TCS Status

Our study identified a set of consistently hypermethylated promoter regions in *H. pylori* 26695 independent of pH or the presence of a functional ArsRS TCS (Table 2). We defined any methylation count greater than three standard deviations above the mean of all promoter regions per methylome as hypermethylated. Thus, each 50 bp promoter region, as defined by Sharma et al. (2010) [34], with six or more methyl marks was classified for the purposes of this study to be hypermethylated. We hypothesize that such strong and consistent methylation suggests potential epigenetic control of the expression of the genes/operons controlled by these promoters.

### 3.6. Hypermethylation of Some Protein Coding Regions Are Influenced by Acidity and Dependent upon the ArsRS TCS

Similar to analyzing hypermethylation in promoters, we applied a methylation frequency threshold of three standard deviations above the mean methylation frequency (measured as the mean of the number of methylated bases within each coding region (CDR) divided by the corresponding length of each CDR) to classify hypermethylation (Table 3).

Our findings reveal 16 hypermethylated CDRs. Two of these hypermethylated CDRs are variable based on ArsRS status. The protein CDR of HP0719, encoding a hypothetical protein, was hypermethylated only in the Δ*arsS* mutant strains at both pH 7 and 5, while that of HP1338, encoding the nickel-responsive regulator (NikR), was hypermethylated only in the control strains, not in the *arsS* deletion mutant. The remaining 14 of these 16 CDRs were hypermethylated independently of culture condition and ArsRS functional status (Table 3).

These observations indicate that certain genes may be selectively methylated depending on the function of the ArsRS TCS. The identification of these hypermethylated regions across different conditions suggests potential epigenetic mechanisms that may affect gene expression in *H. pylori*.

### 3.7. Key Flagellar-Associated, Virulence-Associated, and Outer Membrane Protein Genes Exhibit Differential Methylation Based on pH and ArsRS Functionality

Comparison of the methylomes of the *H. pylori* control mutant revealed 492 unique methylations at pH 7 and 480 unique methylations at pH 5, totaling 972 pH-dependent methylation changes. These sets of methylations are non-overlapping. Of these, 916 occurred within coding regions, four occurred within promoter regions, six overlapped both a coding and promoter region, and 46 occurred within intergenic regions.

Among *H. pylori* 26695 control methylomes, HP0922, a toxin-like outer membrane protein, exhibited the greatest amount of methylation changes, with 11 differential methylations between pH 7 and pH 5 (10 within the coding region and one within the promoter region).

Out of 610 coding regions exhibiting pH-dependent differential methylation, 296 encoded hypothetical proteins, 112 were related to the cell envelope, 72 to translation, and 71 to transport and binding proteins. Of 38 annotated flagellar-associated protein-encoding genes, 16 were differentially methylated within their coding regions when comparing the *H. pylori* 26695 control mutant cultured at pH 7 and pH 5 methylomes. For these genes, 20 unique methylations mapped to the control mutant pH 7 methylome and nine to the control mutant pH 5 methylome (Table 4). Fifteen flagellar-associated protein encoding genes were differentially methylated between the Δ*arsS* pH 5 and pH 7 methylomes. There were 15 methylated positions uniquely within Δ*arsS* pH 7 for these flagellar genes, while there were just four in the Δ*arsS* pH 5 counterparts. The extensive methylation changes in genes encoding cell envelope and motility proteins suggests that *H. pylori* may modify its membrane protein expression, as well as flagellar protein expression, to adapt to temporal variations in the acidic host environments via epigenetic changes.

In the Δ*arsS* mutant, the methylation landscape was less complex. The methylome of the Δ*arsS* mutant cultured at pH 7 possessed 341 unique methylations and 308 when cultured at pH 5 compared to 492 and 480 in the *H. pylori* 26695 control mutant methylomes at these same pH growth conditions. Coding regions displayed 609 methylation differences between Δ*arsS* pH 5 and pH 7, while promoter regions showed only three pH-dependent methylation changes. There were four promoter and coding region methylation differences between the Δ*arsS* mutant methylomes determined for cells cultured at pH 5 and pH 7. Each unique promoter region methylation was found only in the *H. pylori* 26695 Δ*arsS* mutant methylome from cells cultured at pH 7, not from cells cultured at pH 5. These results indicate that some pH-dependent methylations rely on ArsRS, while others are independent of this acid-sensing TCS.

### 3.8. Key Virulence Genes Exhibit Changes in Methylation Patterns in Response to Changing Environmental pH

The *cag* pathogenicity island (*cag*PAI) contains three consistently hypomethylated genes independent of the pH of cultivation and ArsRS functional status, *cag2, cag12,* and *cag15*, discussed above. However, additional methylation differences in *cag*PAI and other virulence-associated genes are evident and dynamic across study conditions. These can be obtained by comparing the methylations in each sample as listed in Supplemental Table S2.

**Figure 3:**
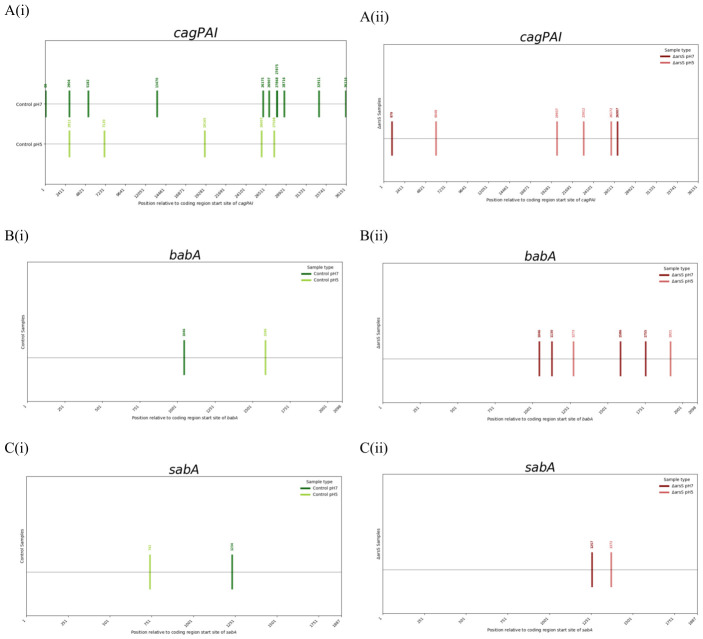
***cagPAI* and the outer membrane protein adhesin-encoding genes *sabA* and *babA* exhibit differential methylation between control and Δ*arsS H. pylori* methylomes at pH 7 and 5.** Panels A–C illustrate methylation positional differences across the four methylomes analyzed: (**A**) the complete cag pathogenicity island; (**B**) *babA* (HP1243); and (**C**) *sabA* (HP0725). Differences unique to individual methylomes are indicated; shared methylation marks are not shown. Each panel labeled (**i**) shows comparisons between control pH 7 and pH 5 samples, with marks denoting sites present in only one of the two conditions. Panels labeled (**ii**) show comparisons involving deletion of the *arsS* gene, where marks indicate methylation sites unique to the deletion mutant at pH 5 or pH 7, respectively.

Fourteen methylation differences were identified across the *H. pylori* 26695 *cag*PAI when comparing control strains cultured at pH 7 and pH 5 (Figure 3A). Two of these differences were within the *cagA* protein coding region. In contrast, only six methylation differences were observed between ∆*arsS* strains at pH 7 vs. pH 5 in *cagPAI*, none of which occurred within *cagA*. This suggests that methylation of *cagA* is dependent on a functional ArsRS two-component system.

*babA* (HP1243), encoding a well-documented outer membrane protein adhesin critical to disease pathogenesis [38], exhibited dynamic methylation changes across our experimental conditions. Comparing the *H. pylori* 26695 control mutants, two methylation differences in *babA* were detected between pH 7 and pH 5. Under acidic conditions, four methylation differences were identified between control pH 5 and ∆*arsS* pH 5. Under neutral conditions, two differences were observed between control pH 7 and ∆*arsS* pH 7 methylomes. Interestingly, position 1586 revealed a pH-dependent and ArsRS-dependent methylation pattern (Figure 3B). Notably, none of these methylation changes corresponded to known motifs, with several identified as m4C modifications. The presence of both m6A and m4C differences across conditions further suggests that additional methyltransferases, including M2.HpyAII (the only known m4C MTase in *H. pylori* 26695), may also contribute to ArsRS-dependent methylation dynamics [15,39,40].

HP0725 (*sabA*), which encodes the sialic acid-binding adhesin protein SabA, also displays ArsRS-dependent methylation dynamics. This gene is transcriptionally regulated by the ArsRS two-component system as well as by phase variation [41,42,43]. Comparing the methylomes of the control mutant, two unique methylation differences exist between pH 7 and pH 5. A similar pattern is found in the ∆*arsS* mutant; however, different nucleotides are methylated than demonstrated in *sabA* in the control mutant methylome (Figure 3C).

### 3.9. Many Genes Differentially Transcribed During Acid Stress Are Also Differentially Methylated in an ArsRS-Dependent Manner

**Figure 4:**
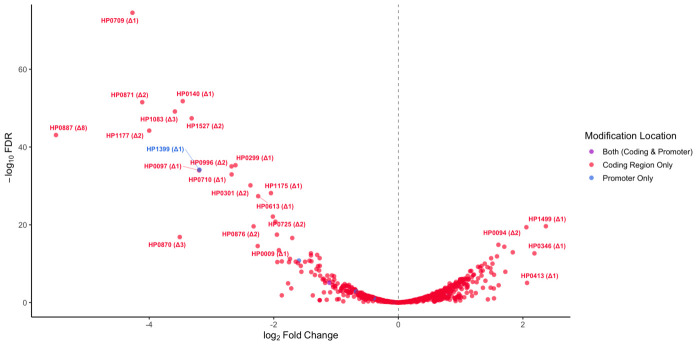
**Volcano plot of differentially transcribed and differentially methylated genes due to changes in acidity.** This plot displays genes that are both differentially transcribed as determined by Loh and colleagues [30] in their 2021 study and differentially methylated between pH 7 and pH 5 conditions, as revealed in our current study. The *x*-axis represents the log_2_ fold change (Log_2_FC) in gene expression, while the *y*-axis shows the statistical significance (-log_10_ *p*-value) of that change in gene expression. Genes with a Log_2_FC > 1 (right side) are upregulated at pH 5, whereas genes with a Log_2_FC < −1 (left side) are downregulated at pH 5, as reported by Loh et al. (2021) [30]. Genes without corresponding differential methylation have been removed. Those differentially expressed genes (DEGs) that are also differentially methylated are labeled with the number of changes in methylation marks (Δ). Those DEGs whose coding regions are differentially methylated are represented as red circles, and those DEGs whose promoters are differentially methylated are shown as blue circles.

The acclimation of *H. pylori* to varying pH environments represents a critical mechanism for the hallmark persistence of this pathogen in the human stomach. In this study, we layered our epigenetic studies upon a 2021 study by Loh and colleagues [30] of the transcriptional responses of *H. pylori* 26695 when exposed to acidic conditions (Figure 4).

Combining our analysis of methylation patterns of *H. pylori* 26695 under pH 7 and pH 5 conditions with data from the study by Loh and colleagues [30], we discovered significant overlaps between transcriptional response and methylomic status of the *H. pylori* 26695 genome under conditions of acidity. Loh et al. found that of 1590 genes analyzed, 170 genes were transcriptionally upregulated and 155 genes were downregulated when the bacteria were grown at a pH 5 for 6 h [30]. We found that 63 of the 170 transcriptionally upregulated genes (37.1%) and 71 of the 155 transcriptionally downregulated genes (45.8%) determined in that study also demonstrated pH-dependent changes in methylation patterns (Figure 4).

**Figure 5:**
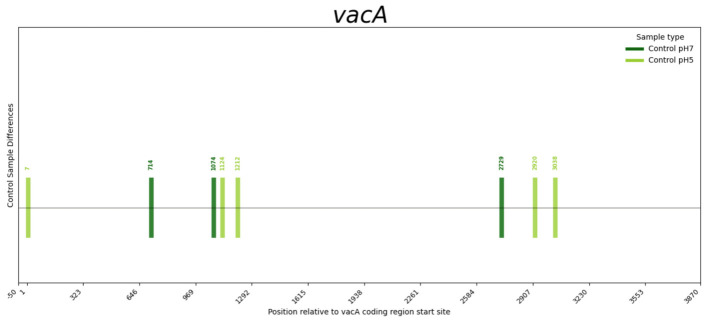
**Differentially methylated positions in *vacA* (HP0887).** Differentially methylated bases in the *H. pylori* 26695 control mutant methylomes cultured at pH 7 and 5. Light green-colored markings correspond to a unique methylation in the control sample at pH 5 which is not present in the pH 7 comparison. Dark green marks are those methylations which are unique in control pH 7. Nucleotide positions are shown relative to the start of the vacA coding region (position 0), with the *x*-axis spanning the full length of the coding region. This shows the start-to-end positions of the vacA protein coding region.

Among the differentially expressed genes, three intriguing candidates emerged as possessing both significant transcriptional and epigenetic modifications. HP0887, encoding the vacuolating cytotoxin, *vacA*, displayed the most dramatic response, being transcriptionally downregulated at pH 5 with a Log_2_FC of −5.5 and encompassing eight differentially methylated positions between control strains pH 5 and pH 7 (Figure 5). The *vacA* coding region in the *H. pylori* 26695 control mutant cultured at pH 7 contained 110 methylation marks, of which three were unique to pH 7, while the same control mutant cultured at pH 5 contained 112 methylation marks, of which five were unique to pH 5. A comparison between these two methylomes revealed eight differentially methylated positions within *vacA* that distinguish the pH 7 and pH 5 conditions.

The promoter of HP1399 (*rocF*), encoding an arginase linked to *H. pylori* virulence [44], showed a log_2_ FC of −3.19 [30] and contained a single methylation site at nucleotide −34 relative to the transcriptional start point present at pH 7 but absent at pH 5 (Figure 4).

HopQ (HP1177), an outer membrane adhesin that promotes CagA translocation via Carcinoembryonic Antigen-related Cell Adhesion Molecule (CEACAM) binding [45,46,47], was transcriptionally downregulated under acidic conditions (log_2_FC = −3.99) and possessed two additional M4C methylation sites within its coding region at pH 7 not seen at pH 5 (Figure 4).

## 4. Discussion

DNA methylation in *Helicobacter pylori* is a key mechanism for modulating gene expression under environmental stress [13,14,15,48]. Despite exposure to fluctuating acidity and robust host immune/inflammatory pressures, this bacterium colonizes the gastric epithelium of over half the world’s population [5,49,50]. Its diverse restriction–modification (R-M) systems not only defend against foreign DNA but also regulate transcription, replication, and genome stability [14,25,51]. Methyltransferases within these systems target specific motifs, shaping gene expression critical for fitness and virulence. As *H. pylori* has a relatively low GC content, the majority of *H. pylori* DNA methyltransferases are N6-adenine methyltransferases rather than cytosine methyltransferases. This bias toward an A+T rich genome creates less opportunities for cytosine methylation [19,52,53].

Variation in R-M systems highlights the genetic plasticity of *H. pylori*. Low-density methylation motifs under selective pressure may enable fine-tuned regulation of traits such as motility, acid resistance, and virulence [54,55,56]. Thus, methylation systems act both as defense mechanisms and as regulators of gene expression. Methylation alters DNA–protein interactions, influencing RNA polymerase binding, transcriptional elongation, and replication [57,58,59]. For example, m5C can lead to C→T mutations through deamination [21,60]. Such changes can also affect mRNA folding and translation efficiency, ultimately shaping protein expression.

Our study revealed distinct methylation patterns throughout the *H. pylori* genome. We observed significant hypermethylation within specific promoter and coding regions. Promoter hypermethylation was conserved across all methylomes analyzed, regardless of culture pH or ArsRS TCS functionality, and may play a regulatory role in the expression of those genes. For example, HP0523, located within the *cag* pathogenicity island (*cag*4), consistently displayed promoter hypermethylation, which may influence expression of this virulence-associated gene independent of environmental pH.

Hypermethylation within coding regions may serve functions beyond transcriptional silencing, such as maintaining genomic integrity or fine-tuning expression levels. Notably, hypermethylated coding regions included HP0559 (acyl carrier protein), which is involved in biosynthesis. This supports a role for methylation in controlling expression during critical metabolic or stress-related states. Additionally, HP1338, encoding the nickel-responsive regulator (NikR), exhibited hypermethylation in the *H. pylori* 26695 control mutant but not in ∆*arsS H. pylori* 26695. NikR has previously been shown to exhibit ArsRS TCS- and pH-dependent transcriptional changes [30]. Such ArsRS-dependent methylation may add another layer of gene expression control to this important transcription factor. The numerous hypermethylated coding regions encoding proteins of unknown function suggest undiscovered epigenetic networks relevant to *H. pylori* biology and pathogenesis.

We also identified a consistently hypomethylated ~21 kbp region across all methylomes examined. This mirrors hypomethylated zones reported in *Campylobacter jejuni* that overlap *oriC* and were linked to virulence [35]. Many genes in this region encode mobile DNA elements, such as transposases and integrases, often associated with genome plasticity and horizontal transfer. The absence of methylation motifs may provide a selective advantage by altering restriction–modification targeting or facilitating expression in novel hosts, complementing the known tendency of mobile elements to be AT-rich [61].

Condition-dependent methylation differences also emerged. Notably, the outer membrane adhesin genes *babA*, *sabA*, and *hopQ*—each transcriptionally downregulated under acidic conditions—showed pH-dependent, ArsRS-independent methylation differences. Such epigenetic flexibility may help *H. pylori* modulate adherence and evade host immune defenses, supporting its well-documented persistent colonization [1,28,30,62,63].

Finally, we detected pH-dependent methylation of the *rocF* promoter near the −35 site, suggesting methylation may regulate expression. *rocF* encodes arginase, a virulence-associated enzyme that hydrolyzes L-arginine into urea and ornithine. Urea fuels the urease pathway, producing ammonia that buffers the periplasmic environment against gastric acid. By depleting L-arginine, *rocF* also limits nitric oxide production by host macrophages, impairing a key antimicrobial defense [44]. The pH-sensitive methylation of *rocF* suggests that *H. pylori* may use epigenetic regulation to fine-tune *rocF* expression based on environmental conditions. Increased methylation at neutral pH may repress *rocF* to conserve energy, while reduced methylation under acidic conditions may enhance *rocF* expression to support acid neutralization and immune evasion. Together, these findings reveal methylation as a dynamic regulatory layer potentially influencing genes critical for *H. pylori* survival and virulence.

In addition to known *H. pylori* adhesin genes, we observed pH-dependent differential methylation within *vacA*, encoding a critical virulence factor noted for its polymorphisms and ability to induce vacuolation in host cells [64,65]. Between control pH 5 and pH 7 methylomes, *vacA* contained eight unique differentially methylated sites and demonstrated a more than 32-fold decrease in mRNA expression under acidic conditions [30]. It is tempting to speculate that this strong repression may be partly epigenetically regulated, suggesting methylation as a control layer for this key virulence determinant and a potential survival strategy in variable gastric environments.

We also identified significant methylation differences across the *cag* pathogenicity island (CagPAI), which encodes the type IV secretion system required for CagA translocation and pro-inflammatory signaling. Multiple pH-dependent methylation changes in both control and Δ*arsS* mutants indicate that DNA methylation may influence CagPAI gene expression, further contributing to *H. pylori* virulence. These findings support a model in which *H. pylori* dynamically adjusts methylation patterns across major virulence genes to adapt and persist.

Strikingly, epigenetic regulation extended to *H. pylori* 26695 genes associated with motility. Of 38 flagellar-associated protein genes annotated in the *H. pylori* 26695 genome [24], 16 were differentially methylated between cells cultivated at pH 7 and pH 5. In the Δ*arsS* mutant, 15 flagellar-associated protein genes were differentially methylated. These extensive shifts suggest that methylation may play a pivotal role in tuning cell envelope structure and motility in response to acid stress, further aiding *H. pylori*’s survival and acclimation to host environmental variability within the gastric niche.

Differential methylation of flagellar genes and adhesins such as *sabA* and *babA* suggests a potential coordinated epigenetic response that modulates *H. pylori* gene expression during environmental acclimation. Reduced methylation of flagellar genes under acidic conditions may affect motility and chemotaxis. Altering motility through methylation of structural genes could shift the bacterium toward altered adhesin expression, promoting stronger attachment to epithelial cells or movement to more favorable niches in response to altered host environmental conditions. This balance between altered motility and adherence may reflect a strategic shift from exploration to colonization, supporting persistence in the gastric environment.

Emerging evidence suggests that *H. pylori*, along with other prokaryotes, may influence host epigenetic landscapes through direct or indirect DNA methylation. A review by Sitaraman et al. [66] discusses potential mechanisms by which prokaryotes, including *H. pylori*, might methylate host DNA, thereby contributing to oncogenic transformation. One proposed mechanism involves the translocation of bacterial DNA methyltransferases into host cells. For example, a HsdM subunit of *Klebsiella pneumoniae* was found to contain a nuclear localization signal and methylated host DNA even in the absence of its specificity subunit HsdS, albeit at reduced efficiency. Similarly, a transposase from *Acinetobacter baumannii* was shown to localize to the nucleus of A549 and COS-7 cells, where it induced specific CpG methylation of the CDH1 (E-cadherin) promoter [67]. Although conclusive evidence is still lacking regarding *H. pylori*, in a 2012 study by Lee et al., 49 of *H. pylori* strain 26695 proteins were found to have predicted nuclear localization sequences and 26 were found to localize to the cell nucleus [68]. Computational predictions by Wang et al. [69] suggest that between one and three of its DNA MTases could potentially be translocated into host cells via the *cag* pathogenicity island (cagPAI) type IV secretion system. Supporting this possibility, studies in Mongolian gerbils demonstrated that *H. pylori* infection leads to increased DNA methylation in gastric epithelial cells [70,71]. While significant investigation is required to confirm these potential mechanisms, if *H. pylori* was capable of translocating MTases into host nuclei and methylating host chromosomal sequences, this may potentially promote altered host gene expression.

Numerous differential methylation patterns were observed in *H. pylori* 26695 between pH 5 and pH 7 controls, as well as between the Δ*arsS* mutants cultivated at pH 7 and pH 5, further suggesting that methylation status may be responsive to environmental changes. Taken together, our results indicate that the ArsRS two-component system shows significant signs of modulating the methylome of *H. pylori* strain 26695. While it is tempting to speculate on the effects of these epigenetic changes documented in our study, much experimental work must be done to examine what effects, if any, these methylome changes generate.

*H. pylori* persistence in the face of host defenses and environmental stress underscores the importance of understanding its regulatory mechanisms, including the emerging phenomena of epigenetic processes such as DNA methylation. Studies on DNA methylation in *H. pylori* may reveal novel mechanisms of gene expression control and thus intervention targets. This regulated epigenetic landscape likely contributes to this pathogen’s persistence, virulence, and various clinical outcomes and represents an underexplored avenue for therapeutic intervention.

## Figures and Tables

**Table 1:** Study design.

Mutation Type	Genetic Modification	pH Condition
Control 1	Δ*rdxA*	7
Control 2	Δ*rdxA*	5
Experimental 1	Δ*rdxA/*Δ*arsS*	7
Experimental 2	Δ*rdxA/*Δ*arsS*	5

**Table 2:** **Promoters hypermethylated regardless of pH conditions and functional ArsRS TCS status.** Primary promoter regions were categorized as 50 base pairs upstream of primary transcriptional start sites defined in Sharma et al. 2010 [34]. Hypermethylated promoter regions with methylation counts exceeding three standard deviations above the mean for primary promoters in each methylome analyzed are listed.

Gene *	Methyl Counts	Start Position	End Position	Function
HP0218	6	227,591	227,641	Hypothetical Protein
HP0352	7	360,631	360,581	Flagellar Motor Switch Protein (*fliG*)
HP0523	6	549,427	549,377	CAG Pathogenicity Island Protein (*cag4*)
HP0985	6	1,048,509	1,048,559	Hypothetical Protein
HP1251	6	1,327,198	1,327,248	Oligopeptide ABC Transporter, Permease Protein (*oppB*)
HP1362	6	1,424,702	1,424,752	Replicative DNA Helicase (*dnaB*)

*—Some of these promoters precede operons with individual downstream genes not listed.

**Table 3:** Hypermethylated protein coding regions across each methylome of *H. pylori* 26695.

Gene	Function	Control pH 7	Control pH 5	Δ*arsS* pH 7	Δ*arsS* pH 5
HP0093	Hypothetical Protein	✓	✓	✓	✓
HP0170	Hypothetical Protein	✓	✓	✓	✓
HP0188	Hypothetical Protein	✓	✓	✓	✓
HP0359	Hypothetical Protein	✓	✓	✓	✓
HP0365	Hypothetical Protein	✓	✓	✓	✓
HP0429	Hypothetical Protein	✓	✓	✓	✓
HP0559	Acyl Carrier Protein (*acpP*)	✓	✓	✓	✓
HP0653	Nonheme Iron-Containing Ferritin (*pfr*)	✓	✓	✓	✓
HP0719	Hypothetical Protein	✗	✗	✓	✓
HP0721	Hypothetical Protein	✓	✓	✓	✓
HP0756	Hypothetical Protein	✓	✓	✓	✓
HP0985	Hypothetical Protein	✓	✓	✓	✓
HP1239	Hypothetical Protein	✓	✓	✓	✓
HP1324	Hypothetical Protein	✓	✓	✓	✓
HP1338	Conserved Hypothetical Protein	✓	✓	✗	✗
HP1531	Hypothetical Protein	✓	✓	✓	✓

**Table 4:** *H. pylori* 26695 flagellar-associated proteins exhibit pH- and ArsRS-dependent differential methylation. * These values note the number of differentially methylated bases when comparing the control mutant at pH 7 and the control mutant at pH 5. ** These values note the number of differentially methylated bases when comparing the Δ*arsS* mutant at pH 7 and the Δ*arsS* mutant at pH 5.

Gene	Gene Name	Control *	Δ*arsS* **
HP0115	Flagellin B (*flaB*)	2	1
HP0295	Flagellin B Homolog (*fla*)	3	1
HP0325	Flagellar Basal-Body L-Ring Protein (*flgH*)		1
HP0351	Flagellar Basal-Body M-ring Protein (*fliF*)	3	1
HP0352	Flagellar Motor Switch Protein (*fliG*)	1	
HP0353	Flagellar Export Protein (*fliH*)	2	
HP0685	Flagellar Biosynthetic Protein (*fliP*)	1	1
HP0751	Polar Flagellin (*flaG*)		
HP0752	Flagellar Hook-Associated Protein 2 (*fliD*)	2	2
HP0815	Flagellar Motor Rotation Protein (*motA*)	2	1
HP0870	Flagellar Hook (*flgE*)	3	2
HP0908	Flagellar Hook (*flgE*)	3	1
HP1031	Flagellar Motor Switch Protein (*fliM*)		1
HP1035	Flagellar Biosynthesis Protein (*flhF*)		1
HP1041	Flagellar Biosynthesis Protein (*flhA*)	1	
HP1092	Flagellar Basal-Body Rod Protein (*flgG*)		1
HP1119	Flagellar Hook-Associated Protein 1 (HAP1) (*flgK*)	2	
HP1274	Paralysed Flagella Protein (*pflA*)	1	2
HP1420	Flagellar Export Protein ATP Synthase (*fliI*)	1	
HP1462	Secreted Protein Involved in Flagellar Motility	1	
HP1558	Flagellar Basal-Body Rod Protein (*flgC*)		1
HP1559	Flagellar Basal-Body Rod Protein (*flgB*)	1	
HP1585	Flagellar Basal-Body Rod Protein (*flgG*)		2

## Data Availability

The code used to generate the figures in this paper is available at https://github.com/skpatterson03/forsyth_lab. URL accessed 28 May 2024. The SMRT sequencing data analyzed in this study were originally published in Zimmerman, E. H. et al. (2024) [12]. The raw and processed data files have been deposited in NCBI’s Gene Expression Omnibus and are publicly accessible through GEO Series accession number GSE241991 (https://www.ncbi.nlm.nih.gov/geo/query/acc.cgi?acc=GSE241991) URL accessed 1 January 2024.

## Supplementary Materials

The following supporting information can be downloaded at https://www.mdpi.com/article/10.3390/microorganisms14071501/s1, Table S1: Unmethylated Promoters. Table S2: Complete Methylomes.
